# Inadvertent defibrillator lead placement into the left ventricle after MitraClip implantation: A case report

**DOI:** 10.1097/MD.0000000000010733

**Published:** 2018-05-11

**Authors:** Giuseppe Santarpia, Francesco Passafaro, Eugenia Pasceri, Annalisa Mongiardo, Antonio Curcio, Ciro Indolfi

**Affiliations:** a Division of Cardiology, Department of Medical and Surgical Sciences, “Magna Graecia” University; b URT-CNR, Department of Medicine, Consiglio Nazionale delle Ricerche, Catanzaro, Italy.

**Keywords:** lead extraction, lead malposition, left ventricle, mitral regurgitation

## Abstract

**Rationale:**

Inadvertent pacemaker/defibrillator lead placement into the left ventricle is an unusual cardiac device-related complication and its diagnosis is not always easy and often misunderstood. Thromboembolic events are frequently associated with this procedural complication. Percutaneous lead extraction should be performed when diagnosis is made early after device implantation while long-life oral anticoagulation is a wise option when the diagnosis is delayed and the lead is not removed.

**Patient concerns:**

A 65-year-old man affected by dilated cardiomyopathy, previously treated with a percutaneous mitral valve repair, with 2 MitraClip devices, and later with dual chamber cardioverter/defibrillator implantation, returned in outpatient clinics 2 months after discharge for deterioration of dyspnea; transthoracic echocardiography revealed that the shock lead had been accidentally placed in the apex of the left ventricle.

**Diagnoses:**

The unintentional lead malposition through the iatrogenic atrial septal defect and its presence into the mitral valve orifice, together with the 2 clip devices implanted, generated an acceleration of transvalvular diastolic flow, determining a moderate stenosis of the mitral valve, as well as promoting a worsening of the degree of valvular regurgitation.

**Interventions:**

Oral anticoagulation therapy was started and a mechanical lead extraction was percutaneously performed. A new defibrillator lead was later appropriately positioned in the apex of the right ventricle.

**Outcomes:**

The patient was discharged 3 days after intervention and the follow-up, performed 1 month after discharge, was uneventful.

**Lessons:**

Complex interventional procedures and implantation of multiple devices can increase procedural troubles and the risk of mechanical complications related to pacemaker/defibrillator implantation. Careful observation of the QRS complex morphology on the electrocardiogram (ECG), during paced rhythm, and the achievement of the echocardiographic examination, in the postprocedural phase, allow an early diagnosis of lead malposition.

## 1 Introduction

Inadvertent pacemaker/defibrillator lead placement into the left ventricle (LV) is an unusual cardiac device-related complication.^[1]^ It is associated with thromboembolic events^[2]^ and it is often, in the current clinical practice, an under-diagnosed condition. The management of this complication is represented by percutaneous lead extraction, when diagnosis of inadvertent lead malposition is made early after device implantation. In fact in this stage fibrosis and vascular adhesions of the lead are not considerable; therefore, its removal can be, safely, carried out and significantly reduce the risk of future thromboembolic events. On the other hand, when diagnosis of lead malposition is delayed and lead adhesions are conspicuous, its removal may be difficult and complicated by systemic embolization; therefore in these cases long-life oral anticoagulation can be reasonably considered a valid alternative therapy to lead extraction.^[3]^

## 2 Case presentation

**Figure 1:**
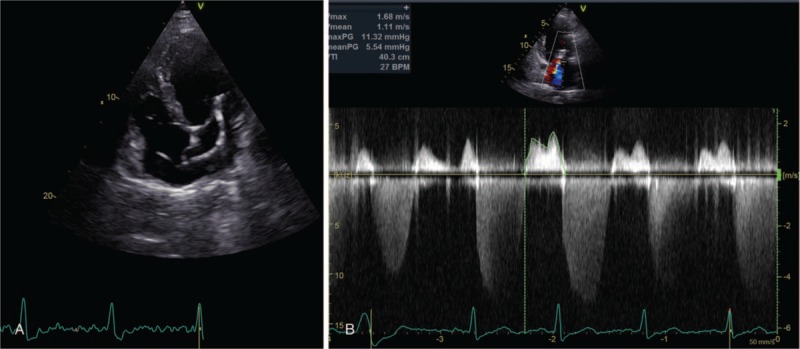
A, Four-chamber 2D echocardiographic view showing defibrillator lead placed in the left ventricle after its passage through an iatrogenic atrial septal defect. B, Echocardiographic 2D Doppler view displaying an accelerated transmitral diastolic flow. 2D = two-dimensional.

**Figure 2:**
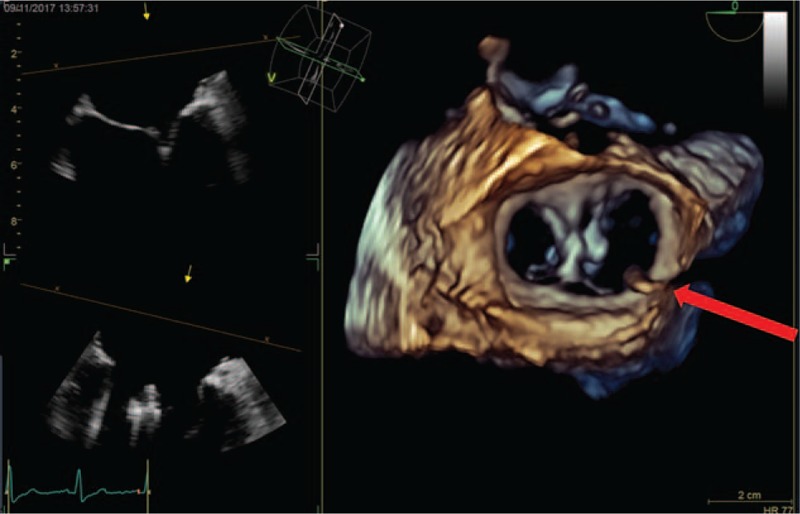
Short-axis left ventricular transesophageal 3D echocardiographic view showing the transit of the defibrillator lead across atrial septal defect (red arrow) and documenting a noncontinuity-solution between defibrillator lead and mitral clips. 3D = three-dimensional.

**Figure 3:**
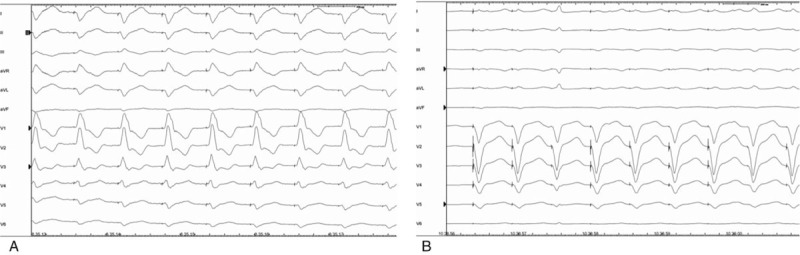
A, Twelve-lead ECG, performed before extraction procedure showing right bundle branch block morphology of the QRS complex, during ventricular pacing. B, Twelve-lead ECG, performed after lead replacement in the apex of the right ventricle, showing a left and superior direction of the QRS complex axis, during ventricular pacing. ECG = electrocardiogram.

**Figure 4:**
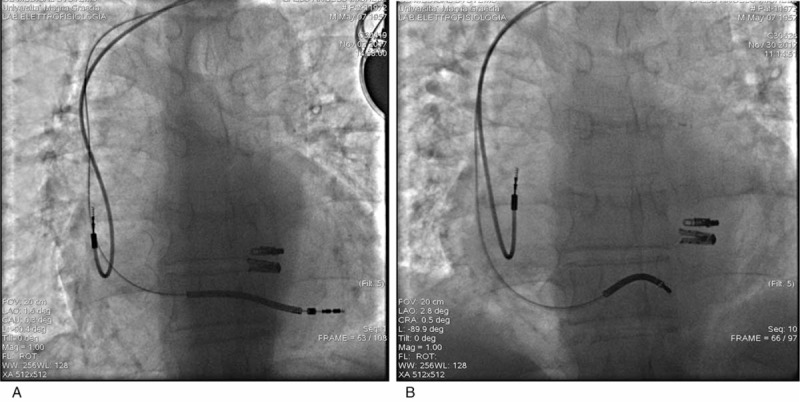
A, Anteroposterior radiographic view showing defibrillator lead in the left ventricle. B, Antero-posterior radiographic view showing defibrillator lead replaced in the apex of the right ventricle after extraction procedure.

We report the case of a 65-year-old man affected by dilated cardiomyopathy associated with severe mitral valve regurgitation, recently treated by 2 MitraClip System devices (Abbott Vascular, Lake Bluff, IL) implantation. Three months after valve repair, the patient underwent to dual-chamber defibrillator implantation because the persistence of seriously dilatation and marked systolic impairment of the LV (ejection fraction = 30%) with mild mitral valve regurgitation. The patient performed a predischarge electronic check of the device that showed its normal function. He came to our observation, 2 months later, for marked deterioration of the dyspnea, despite his strict compliance to medical therapy. At this time, the 2-dimensional (2D) echocardiographic examination showed malposition of the shock lead that appeared placed in the apex of the LV through an iatrogenic atrial septal defect (ASD) persisted after the percutaneous valve repairing procedure, previously performed (Fig. 1A); the LV appeared markedly dilated and presented a severe systolic dysfunction (ejection fraction = 30%). The echocardiographic color-Doppler view showed a moderate mitral valve regurgitation and a significant mitral valve stenosis attested by an accelerated transvalvular diastolic flow (Fig. 1B). The transesophageal 3-dimensional-echocardiographic short axis view on mitral annulus showed a normal implantation of the MitraClip devices on the edges of mitral valve leaflets and a noncontinuity solution between MitraClip devices and the defibrillator lead (Fig. 2). The 12-lead standard electrocardiogram (ECG) showed normal sinus rhythm to 67 bpm and its record during the pacing threshold test bared a right bundle branch block (RBBB) pattern of the QRS complex (Fig. 3A). Chest X-ray documented a very lateral position of the defibrillator lead (Fig. 4A). Oral anticoagulation therapy with adjusted-dose warfarin was directly started and, after obtaining the patient's informed consent, a mechanical lead extraction with subsequently lead repositioning in the right ventricle (RV) was performed 3 weeks later. The patient was discharged in good clinical condition and the 12-lead ECG and the chest X-ray, performed in the predischarge phase, confirmed the right position of the defibrillator lead, respectively, showing a left and superior direction of the QRS complex axis, during ventricular pacing (Fig. 3B) and a conventional position of the lead in the apex of the RV (Fig. 4B). The predischarge echocardiographic examination showed left ventricular systolic dysfunction (ejection fraction 34%) with mild mitral valve regurgitation and a not relevant velocity of trans-mitral diastolic flow. The follow-up performed 1 month after discharge was uneventful. Ethical approval for this paper was not required by the Ethics Committee of our institute, as it is a case report. The patient provided informed consent for this report and his medical data have been anonymized.

## 3 Discussion

Inadvertent malposition of pacing/defibrillator leads into the LV that is a rare complication of the pacemaker/defibrillator implantation. The accidentally passage of the lead through an ASD or a patent foramen ovale is the most common reported cause.^[4–6]^ Lead malposition into LV has also been reported in the setting of ventricular septal or apical perforation and in case of accidental subclavian artery cannulation;^[3]^ risk factors are usually represented by abnormal thorax anatomy and operator inexperience.^[1]^ In the case above described, the crossing point was represented by an iatrogenic ASD persisted after a percutaneous mitral valve repair intervention. The diagnosis of this condition is not always easy and often misunderstood. RBBB pattern on ECG during ventricular pacing is the most useful sign to trigger the diagnostic suspicion of inadvertent pacing lead placement in the LV. This pattern shows a good sensitivity but a poor specificity for LV lead malposition, since it may be seen in other conditions such as RV dilatation or septal pacing.^[7]^ Unfortunately, during a routine electronic check of the device, a 12-lead-ECG during ventricular stimulation is rarely recorded, especially if the patient shows a spontaneous nonstimulated ventricular rhythm. Electronic parameters are not useful in the diagnostic process, because they can also be normal. Chest X-ray is useful to indicate the position of the ventricular lead in the procedural phase and can be used as a confirmatory test in suspected lead malposition, together with 2D- or 3D echocardiography or cardiac computed tomography scan. In this case, the patient presented a spontaneous nonstimulated ventricular activation that concealed the RBBB stimulation pattern, the electronic parameters were perfectly normal and the X-ray imaging of LV-placed and repositioned RV-placed lead showed similar images. Such unfavorable combination of features could explain the missed diagnosis in the predischarge phase. The diagnosis of inadvertent LV lead implantation is often casual, and it is made difficult considering that the patient is often asymptomatic. Conversely, in the described case, the malposition was suspected because the patient showed a worsening of dyspnea. Probably, the presence of the lead into the mitral valve orifice, together with the 2 clip devices implanted, could have generated an acceleration of transvalvular diastolic flow determining a moderate stenosis of the mitral valve, as well as promoting a worsening of the degree of valvular regurgitation. The most dangerous risk of LV lead implantation is thromboembolism resulting from thrombus formation around the site of lead placement, occurring from few days up to several years after the implantation procedure in approximately 40% of the patients.^[8]^ Thus, the initiation of anticoagulation therapy is mandatory to prevent thromboembolic complications, independently of symptoms, or imaging evidence of thrombosis, since in a series of surgery explanted leads, transthoracic and transesophageal echocardiography failed to correctly identify thrombus formation preoperatively.^[2]^ Therefore, we decided to anticoagulate the patient, even if he was assuming aspirin 100 mg and clopidogrel 75 mg once day, since thromboembolism has been described in several patients receiving antiplatelet therapy,^[9]^ while the incidence of thromboembolic events on anticoagulation therapy was very low.^[3]^ There are different strategies to manage an inadvertent LV lead implantation. Time from implantation to diagnosis is the main factor influencing the choice. Percutaneous lead extraction is the best option since this strategy can reduce the risk of thromboembolic events without the need for lifelong anticoagulation therapy. This procedure carries a risk of systemic embolization and its success rate is acceptable only in patients who received an early diagnosis. If the diagnosis of inadvertent LV lead placement is delayed, long-life anticoagulation with warfarin is reasonable considering the very low risk of thromboembolic event in anticoagulated patients.^[3]^ Surgical LV lead extraction, especially when cardiac surgery is indicated for other clinical reasons, should be considered in patients with a firm indication to lead extraction and a late diagnosis or with a clearly contraindication to the percutaneous procedure. Surgical lead extraction may be preferable in younger patients in whom the risk of surgery is lower and the planned duration of anticoagulation therapy is longer, and can be performed, nowadays, with a minimally invasive approach.^[10]^ In the reported case, the diagnosis was made about 3 months after implantation. The patient was symptomatic and presented a high baseline thromboembolic as well as surgical risk. Furthermore, despite the contiguity of the defibrillator lead with the mitral repairing clip devices, the lack of a direct continuity relationship between them has led us to hypothesize a low risk of MitraClips dislocation during a potential percutaneous mechanical lead extraction procedure. Thus we decided to extract the lead in this way, with a complete procedural success and a clear clinical benefit.

## 4 Conclusion

The case above described demonstrated that complex interventional procedures and implantation of multiple devices can increase procedural troubles and the risk of mechanical complications related to pacemaker/defibrillator implantation. Careful observation of the QRS complex morphology on the ECG, during paced rhythm, and the execution of the echocardiographic examination, in the postprocedural phase, allow an early diagnosis of lead malposition, encouraging the revision of the pacemaker/defibrillator system in the early stage, reducing the risk of future thromboembolic strokes.

## Author contributions

**Conceptualization:** Giuseppe Santarpia.

**Data curation:** Eugenia Pasceri.

**Methodology:** Eugenia Pasceri.

**Project administration:** Giuseppe Santarpia, Francesco Passafaro.

**Supervision:** Annalisa Mongiardo, Ciro Indolfi.

**Validation:** Giuseppe Santarpia, Annalisa Mongiardo, Ciro Indolfi.

**Visualization:** Giuseppe Santarpia, Antonio Curcio.
